# PANOMICS meets germplasm

**DOI:** 10.1111/pbi.13372

**Published:** 2020-05-19

**Authors:** Wolfram Weckwerth, Arindam Ghatak, Anke Bellaire, Palak Chaturvedi, Rajeev K. Varshney

**Affiliations:** ^1^ Molecular Systems Biology (MOSYS) Department of Functional and Evolutionary Ecology Faculty of Life Sciences University of Vienna Vienna Austria; ^2^ Vienna Metabolomics Center (VIME) University of Vienna Vienna Austria; ^3^ Center of Excellence in Genomics & Systems Biology International Crops Research Institute for the Semi‐Arid Tropics (ICRISAT) Hyderabad Telangana India

**Keywords:** Green systems biology, PANOMICS, plant systems biology, multi‐omics, phenotyping, crop improvement, germplasm, genome editing, GWAS

## Abstract

Genotyping‐by‐sequencing has enabled approaches for genomic selection to improve yield, stress resistance and nutritional value. More and more resource studies are emerging providing 1000 and more genotypes and millions of SNPs for one species covering a hitherto inaccessible intraspecific genetic variation. The larger the databases are growing, the better statistical approaches for genomic selection will be available. However, there are clear limitations on the statistical but also on the biological part. Intraspecific genetic variation is able to explain a high proportion of the phenotypes, but a large part of phenotypic plasticity also stems from environmentally driven transcriptional, post‐transcriptional, translational, post‐translational, epigenetic and metabolic regulation. Moreover, regulation of the same gene can have different phenotypic outputs in different environments. Consequently, to explain and understand environment‐dependent phenotypic plasticity based on the available genotype variation we have to integrate the analysis of further molecular levels reflecting the complete information flow from the gene to metabolism to phenotype. Interestingly, metabolomics platforms are already more cost‐effective than NGS platforms and are decisive for the prediction of nutritional value or stress resistance. Here, we propose three fundamental pillars for future breeding strategies in the framework of Green Systems Biology: (i) combining genome selection with environment‐dependent PANOMICS analysis and deep learning to improve prediction accuracy for marker‐dependent trait performance; (ii) PANOMICS resolution at subtissue, cellular and subcellular level provides information about fundamental functions of selected markers; (iii) combining PANOMICS with genome editing and speed breeding tools to accelerate and enhance large‐scale functional validation of trait‐specific precision breeding.

## Introduction

Climate change and food security are the two major issues of the 21st century. It is well estimated that by 2050, the world population will reach ~9 billion and ~9–11 billion by 2100. The current practice of food production using conventional breeding approaches is insufficient to satisfy the global demand by 2050, especially with the view to agroecological questions such as productivity, robustness, sustainability and equitability. Moreover, agricultural productivity and environmental changes are well correlated; any abrupt change in environmental conditions will have a consequent harsh impact on plant productivity owing to the direct and indirect impact of biotic and abiotic stresses (Lobell and Gourdji, 2012). Due to these changing events, breeders and plant scientists are under pressure to exploit and improve existing germplasm and develop new high‐yielding crops that are more nutritious, resistant and climate‐resilient (Long *et al.*, 2015).

**Figure 1:**
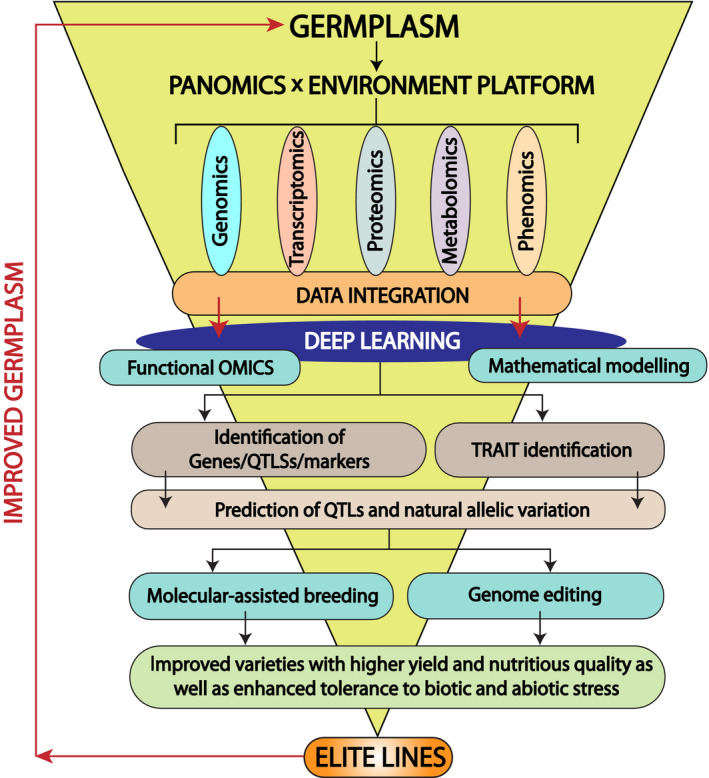
PANOMICS to germplasm. Data from PANOMICS subfields are integrated to guide breeding processes in order to provide elite lines to improve germplasm.

Plant breeding is a co‐evolutionary process. For instance, domestication, the most primitive form of plant breeding, began around 10–12 thousand years ago, people around the world explored and cultivated around 7000 edible plant species from the wild ancestors (Hancock, 2005). Subsequently, the discovery of Mendel’s law initiated new tools for plant breeding based on a genetic crossing. Gartons Agricultural Plant Breeders was established in the 1890s by John Garton, who was one of the first to commercialize new varieties of crops created through cross‐pollination. Initially, plant breeding methods were mainly based on the phenotypic selection, and it was very effective for the traits with simple genetic make‐up, for example traits with higher heritability (Hallauer *et al.*, 2010), but it was time‐consuming and labour‐intensive. However, the ultimate goal of plant breeding is to achieve the genetic gains for desirable traits in time‐ and cost‐efficient manner. Therefore, traditional breeding techniques are no longer sufficiently powerful to satisfy current and future needs of food security and sustainability (Langridge and Fleury, 2011). Moreover, crop productivity is governed by several complex traits manifested by genetic and epigenetic interactions (e.g. genetic correlation between the traits). The approach of molecular markers has considerably advanced and provides an opportunity for genotypic selection where the genetic location of key loci is known. Several molecular breeding approaches including marker‐assisted selection/marker‐assisted backcrossing, marker‐assisted recurrent selection and genomic selection have been used to speed up the breeding process. However, these strategies have limitations that can complicate breeding efforts, for example lethal alleles, redundant genes and their functions. These approaches always consider genes as independent functional entities and hence perform well when the targeted agronomical trait is controlled by one or few genes, which is not the case for traits with complex multigenic regulation and strong environmental dependency such as drought resistance. Each gene can change its function in different environmental conditions, and this is not predictable from the genome sequence alone but rather from causal molecular processes under a plethora of different environmental pressures. This information can be undetected by forward and reverse genetics. Hence, it is of utmost importance to integrate all molecular levels of a biological system that reflects the complete information flow from the gene to metabolism to phenotype in the environmental context which was proposed in the framework of Green Systems Biology (Weckwerth, 2003; Weckwerth, 2011). This opens up a completely new perspective for plant breeding. Here, we propose a ‘PANOMICS platform’ that statistically and mathematically integrates complex ‘‐omics’ data sets arising from genomics, epigenomics, transcriptomics, proteomics, post‐translational modification (PTM) proteomics, metabolomics and phenomics. The PANOMICS platform is expected to facilitate crop improvement by discovering target genes and pathways for physiological phenotypes that are controlled by complex genetic and epigenetic mechanisms with the ultimate goal of ‘precision breeding’ to produce elite lines (Figure 1). This comprehensive information about the molecular system can be integrated through powerful data mining techniques (Weckwerth, 2011; Weckwerth, 2019).

Based on these high‐throughput technologies, we propose three fundamental pillars for future breeding strategies: Strategy 1: combining genome selection with environment‐dependent PANOMICS analysis and deep learning to improve prediction accuracy for marker‐dependent trait performance (Figure 1); Strategy 2: PANOMICS resolution at subtissue, cellular and subcellular level provides information about fundamental functions of selected markers; Strategy 3: combining PANOMICS with genome editing and speed breeding tools to accelerate and enhance large‐scale functional validation of trait‐specific precision breeding.

In this review, we highlight the progress achieved in understanding the PANOMICS platform and their integration into new breeding strategies.

## Yield defining traits and opportunities for germplasm improvement

**Figure 2:**
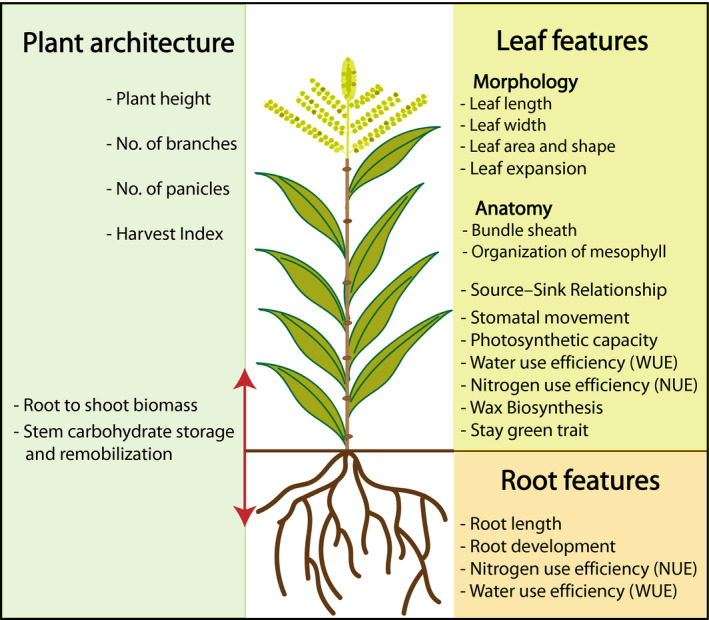
Plant morphological and physiological traits relevant for high yield.

Agricultural productivity is strongly associated with plant growth and development. Several developmental and physiological features such as plant architecture (plant height, number of panicle, number of branches, root length, shoot to root biomass, etc.), leaf features (morphology, anatomy, stomatal movement, leaf growth rate, stay green trait, etc.), carbon use efficiency (CUE), nitrogen use efficiency (NUE) and water use efficiency (WUE) determine major traits that contribute for overall crop performance and yield (Figure 2). For example, leaf features determine the quantity of light interception, photosynthetic capacity and direct mobilization of photosynthates from source to sink that are crucial for efficient partitioning of photoassimilated carbon (Horton, 2000). Similarly, nitrogen (N) is an important constituent. It comprises 1.5%–2% of plant dry matter and ~16% of total plant protein. It is also an important factor in controlling crop yield and grain protein content (Kant *et al.*, 2012). However, in order to enhance agricultural productivity, excessive nitrogenous fertilizers are used which has now become a potential environmental threat leading to lethal nitrification. Overall assessment of NUE in plants comprises both uptake and utilization efficiencies. It is significant to increase NUE of crops to minimize the loss of N and decrease environmental pollution. However, suppression of soil nitrification has been observed to occur naturally in some ecosystems and is termed as biological nitrification inhibition (BNI), indicating that the inhibition originated from plants in the ecosystem (Subbarao *et al.*, 2013; Figure 2). Therefore, for sustainable agricultural productivity, the optimization of these important features is extremely important.

Opportunities for crop improvement include engineering of these complex traits to improve yield. This requires a thorough understanding of the genetic system and information flow from gene, RNA, protein and metabolite to these traits. Furthermore, developing ecophysiological models by integrating the physiological traits with the PANOMICS approach can improve prediction accuracy and marker identification in the environmental context. Considerable progress has been made in deciphering the genetic and molecular basis of the developmental processes that govern these traits, particularly in the model plant species *Arabidopsis thaliana*. However, such a detailed understanding of crop plants is rather at its infancy.

## PANOMICS platform for germplasm improvement

### Genomics: era of Big Data

The application of genomics in the field of plant breeding started with the advent of RFLP marker technology (Tanksley *et al.*, 1989). However after plant genome sequence assemblies started to be available, a combination of conventional breeding techniques with genomics tools and approaches led to a new concept of ‘genomics‐assisted breeding’ (Varshney *et al.*, 2005). Genomics provides breeders with a new set of tools and techniques to study and understand the whole genome and associate relationship of genomic segments with the phenotype. This association can be harnessed by breeders to enhance selection efficiency and precision in plant breeding for the development of efficient populations with high yield and quality (Varshney and Dubey, 2009).

Owing to the high cost of the Sanger method (Sanger *et al.*, 1977), initially, genome sequencing was restricted to microorganisms and species with typically small genomes, e.g. in 2001, sequencing of the first plant model species *Arabidopsis thaliana* was at a cost of approximately $100 million followed by rice in 2005 (Kaul *et al.*, 2000; Matsumoto *et al.*, 2005). With the subsequent development of ‘next‐generation’ sequencing (NGS) technologies and platforms (viz. Illumina, ABI SOLiD, PacBio sequencing, Nanopore single‐molecule sequencing from Oxford Nanopore Technologies, MinION), the costs of sequencing were reduced by several orders of magnitudes. As a result, genomes of several hundreds of plant species have been assembled for understanding genome architecture, genome variations in germplasm collections that may be linked with climate change and agronomy‐related traits in plant breeding.

Genomics is widely used for germplasm enhancement with two overarching aims (a) to enhance breeding lines and (b) improve genetic stocks. Genetic stocks represent new types of germplasm collections that include mutants generated using DNA insertion (Sallaud *et al.*, 2004), physical and chemical mutagenesis (Waugh *et al.*, 2006), recombinant inbred lines (RILs), double haploid lines (DHs), introgression lines (ILs), near‐isogenic lines (NILs), nested association mapping (NAM) population (McMullen *et al.*, 2009) and multiparent advanced generation intercross (MAGIC) populations (Cavanagh *et al.*, 2008). All these enhanced germplasm collections are extremely important for genetic mapping, cloning and functional genomics research. For example, the MAGIC population is exploited for developing populations with large phenotypic diversity, which can be useful for high‐resolution QTL mapping (Kover *et al.*, 2009). Another important example is the successful release of submergence tolerant rice verities that included fine mapping of SUBMERGENCE 1 (SUB 1) locus from FR13A. Using marker‐assisted backcrossing (MABC), the SUB1 region was introgressed into modern high‐yielding varieties of rice (Bailey‐Serres *et al.*, 2010).

## Genomic selection and prediction tools for germplasm improvement

Genomics largely facilitates molecular breeding that is majorly performed by two approaches: marker‐assisted selection (MAS; Ribaut and Hoisington, 1998) and genomic selection (GS) (Meuwissen *et al.*, 2001). Marker‐assisted selection (MAS) tool has been successfully applied in almost all the crop breeding programmes. Here, the small number of genes or traits are identified using linked DNA marker at an early stage before the production of the next generation, thus facilitating the improvement of traits that cannot be improved by the conventional breeding process. Genomic selection (GS), by contrast, uses genome‐wide DNA markers to predict the genetic merit of the complex traits employed for breeding (Desta and Ortiz, 2014; Meuwissen *et al.*, 2001), for example understanding complex trait such as yield that is affected by variants in a large number of genes and regulatory elements. The effect of these variances can easily be captured through trait mapping considering linkage disequilibrium (LD) with genome‐wide DNA markers for single nucleotide polymorphisms (SNPs). The effect of these markers is estimated over large populations and trait is measured. Once the molecular marker (DNA) linked to the trait is identified, candidate lines for breeding can be selected. Further, the identified candidate lines are assessed by genomic breeding value (GEBV); it is a statistical model that considers the sum of effects for marker alleles that each candidate line carries. The lines with the highest GEBV value are selected for breeding the next‐generation crops (Crossa *et al.*, 2017; Desta and Ortiz, 2014). Genomic selection is being popularly used in the breeding programmes; for example, the maize breeding programme ‘AQUAmax’ generated a drought‐tolerant maize hybrid variety by genomic selection that has significantly higher yields under drought stress condition (Gaffney *et al.*, 2015). Therefore, to achieve higher genetic gains, multiple traits must be targeted simultaneously using genomic selections.

Some of the widely used prediction models for genomic selection include parametric models like genomic best linear unbiased prediction (GBLUP; Habier *et al.*, 2013; VanRaden, 2008); ridge regression BLUP (rrBLUP; Endelman, 2011); the least absolute shrinkage and selection operator (LASSO; Usai *et al.*, 2009); the elastic net (EN; Zou and Hastie, 2005); Bayesian ridge regression (BRR; Gianola *et al.*, 2003); Bayesian least absolute shrinkage and selector operator (BL; Park and Casella, 2008); and BayesA, BayesB and BayesC (Habier *et al.*, 2011). In addition, the following nonparametric models include reproducing kernel Hilbert space regression (RKHS) (Campos *et al.*, 2010), support vector machine (SVM; González‐Recio *et al.*, 2014), relevance vector machine (RVM; Tipping, 2001), Gaussian Processes (GP; Williams, 1998) and random forest (RF). GP is often used in machine learning to predict the value for an unseen point in the training data and defined as a collection of random variables (Rasmussen and Williams, 2004). These statistical models are used extensively to predict unobserved individuals in genomic selection for germplasm enhancement. For example, solGS, which is a Web‐based tool for predicting phenotypic correlation, heritability of traits and selection indices of individuals, is based on the rrBLUP model (Tecle *et al.*, 2014).

To enhance the returns from genomic selection, systematic genetics can be combined with other technologies like transcriptomics, proteomics and metabolomics to explore complex traits (PANOMICS approach). This information improves the understanding of causal processes and the prediction accuracy for genomic selection (Luo, 2015; Schrag *et al.*, 2018; Weckwerth, 2011; Zamir, 2001). In addition, allele mining can also be used to identify superior alleles (Barkley and Wang, 2008). For example, EcoTILLING is a well‐established, cost‐effective approach, which is used to identify novel alleles for genes associated with genes controlling agronomic traits in diverse germplasm (Yu *et al.*, 2012). However, it is necessary to uncover the function of the candidate gene with agronomically valuable loci and their potential implementation in genome editing (see section on PANOMICS‐guided genome editing for precision breeding) to accelerate germplasm improvement.

## Transcriptomics: from microarrays to next‐generation sequencing (NGS)

Transcriptomics is the study of gene expression, and this area of research was greatly facilitated due to the establishment of EST sequencing projects in major plant species (Sreenivasulu *et al.*, 2002) in the late 1990s. It is a widely used method to measure all mRNA transcripts in one cell or a population of cells (Wang *et al.*, 2009). Though genomics provides sequence information, the study of transcriptomics is necessary because (a) in transcription conditions not all genes are expressed simultaneously throughout plant growth and development and (b) other classes of RNA such as miRNA, snoRNA and sRNA cannot be studied using genomic tools. Moreover, due to the homogenous and simple structure of RNA, transcriptome profiling is rather straight forward compared to proteomics and metabolomics.

In order to efficiently use transcript profiling to identify the specific gene involved in the trait, it is essential to combine it with genetic or QTL mapping and this procedure is referred as ‘genetical genomics’ or expression genetics (Varshney *et al.*, 2005). In this approach, total mRNA or cDNA of the organ/cell/tissue from each individual mapping population is hybridized onto a microarray carrying a high number of cDNA fragments representing the species/tissue of interest and quantitative data are recorded reflecting the level of expression of each gene on the filter (de Koning and Haley, 2005). Further assuming, that every gene showing transcriptional regulation is mapped within the genome of the species of interest, the expression data can be subjected to QTL analysis, thus making it possible to identify the so‐called ‘ExpressQTLs’ (eQTLs). eQTL analysis identifies gene products influencing the quantitative trait (level of mRNA expression; Schadt *et al.*, 2003). RNAseq analysis of wheat root tissue under drought stress led to the identification of 45139 DEGs, 13820 TF, 288 miRNAs, 640 pathways and 435829 putative markers (28807 SSRs, 276369 and 130653) variants in two contrasting genotypes. Further analysis revealed the drought‐responsive QTLs on chromosome 3B in wheat roots possess 18 differentially regulated genes with 190 sequence variants (173 SNPs and 17 InDels; Iquebal *et al.*, 2019). Similarly, transcriptome analysis of wheat root tissue revealed up regulation of auxin receptor (AFB2) and ABA‐responsive transcription factors (MYB78, WRKY18 and GBF3) under drought stress (Dalal *et al.*, 2018).

Furthermore, specialized NGS technologies are now facilitating scRNA (single‐cell RNA) sequencing that can enable a clear understanding of distinct cell identities and transition states and it also provides evidence about the unique mutations in the cell (Bokszczanin *et al.*, 2015; Shapiro *et al.*, 2013). Plant tissues and cells are highly specialized morphologically, biochemically and physiologically (Nelson *et al.*, 2008). scRNA sequencing confers the ability to quantitate and identify RNA molecules specific to a particular cell population. Fricke and co‐workers demonstrated the ion and metabolite distribution of individual epidermis cells in barley leaf, and this distribution depends upon the developmental stage of leaf (Fricke *et al.*, 1994). In this study, two main purposes of single‐cell analysis were highlighted, understanding the individuality of cell stages, and their differential response to environmental stimuli. scRNA studies have also successfully described the development and differentiation of other unique plant morphologies, such as stomatal cells (Adrian *et al.*, 2015), pollen (Bokszczanin *et al.*, 2015; Honys and Twell, 2003) and female gametophytes (Schmid *et al.*, 2015). Here, we propose that combining scRNA sequencing with genome editing technologies can boost crop improvement and support precision breeding because CRISPR‐seq and related techniques rely on a guide RNA (gRNA) vector with a unique barcode that can be detected in scRNA sequencing (Datlinger *et al.*, 2017).

In addition, due to the rapidly growing accumulation and diversity of identified RNA sequences, further development of computational models will be required to analyse, interpret and integrate these data in order to potentially use them for precision breeding.

## Proteomics: proteins are doing the job

For precision breeding, it is of utmost importance to harness the functional units from the sequenced genomes. Gene function can only be defined in the context of the corresponding protein and its isoforms because they are the active molecular entity in an organism. Accordingly, the interpretation of genome functions is only valid when the spatial and temporal activities and interactions of the proteins are well characterized (Salekdeh and Komatsu, 2007). Genes are transcribed into mRNA using alternate splicing and transcripts are translated into proteins, but it is often observed that mRNA levels do not well correlate with protein abundance. Therefore, it is important to study and understand proteins that are translated from the genes because consequently, one gene can produce several different protein isoforms.

A proteome has dynamic capabilities, unlike the genome. The study of proteins reveals the functional players that are mediating specific cellular processes. Further, proteomic studies also focus on post‐translational modifications (PTMs), subcellular localization and compartmentalization, protein complexes, signalling pathways and protein–protein interactions, all this not predictable from the genome sequence (Chaturvedi *et al.*, 2016; Ghatak *et al.*, 2017b). Therefore, parallel development of various advanced bioinformatics and computational tools is needed in order to integrate proteomics to other ‘‐omics’, and the physiological data that can further open up new methods for crop improvement studies (Kitano, 2002; Langridge and Fleury, 2011). The most widely used proteomics methods are the protein‐based approach (gel‐based approach for two‐dimensional electrophoresis (2‐DE)) and peptide‐based approach (gel‐free or shotgun proteomics approach) (Chaturvedi *et al.*, 2015; Chaturvedi *et al.*, 2016; Chaturvedi *et al.*, 2013). Technological advances have also allowed to explore targeted MS‐based quantitative approaches, which include selective reaction monitoring (SRM), multiple reaction monitoring (MRM), parallel monitoring reaction (PRM) and accurate inclusion mass screening (AIMS; Boersema *et al.*, 2015; Borras and Sabido, 2017; Gillet *et al.*, 2016; Lehmann *et al.*, 2008; Picotti and Aebersold, 2012; Wienkoop *et al.*, 2008; Wienkoop and Weckwerth, 2006; Wienkoop *et al.*, 2010). These are powerful techniques for the identification of specific proteins with causative functions in an agronomically important trait and do allow for high sample throughput (Jacoby *et al.*, 2013). These techniques have been explored mainly in the laboratory under highly controlled growth conditions. The major question is whether, for example, shotgun proteomics can also be applied in field studies under ‘native’ growth conditions for staple food crops. In 2008, we performed one of the first studies of large proteomics screening in field trials of potato breeders (Hoehenwarter *et al.*, 2008). By implementing a novel rapid data processing and mining approach of these high‐dimensional proteomics data including machine learning, it was possible to identify novel and robust protein markers predicting specific traits in potato such as starch content and black spot disease susceptibility. Furthermore, we were able to use the mass spectrometric data to identify protein polymorphisms that were not predicted by available databases. We applied the approach called PROTMAX for the characterization of several hundreds of field measurements for various potato genotypes (Hoehenwarter *et al.*, 2011b). Here, polyploidy is a severe problem because of the multiplication of protein isoforms in the genome and their potential functional diversification. We addressed this problem with the combination of shotgun proteomics and linear mathematics to resolve protein isoforms (Hoehenwarter *et al.*, 2011a). We will address these questions in more detail in the future.

From an agricultural perspective, seed viability is the most important factor for crop production. Proteomics studies in seed development elucidate the molecular pathways and physiological transitions, which can contribute to the advancement of valuable and potentially agriculturally important strategies for improving yield, quality and stress tolerance in crop plants (He and Yang, 2013). For example, analyses of seed protein content and to understand the role of enzymes involved in the starch biosynthesis aid plant breeders to analyse genes responsible for seed quality and to generate predictive hypotheses. In the study performed by Komatsu and Hossain, proteomics was used to investigate the regulation of rice seed germination which revealed the detailed mechanism of starch degradation in endosperm and starch biosynthesis in the embryo during seed development (Komatsu and Hossain, 2013). Recently, the shotgun proteomics approach on mature barley seeds enabled a more complete characterization of the barley seed proteome (Mahalingam, 2017). In this study, a key difference in hordoindoline (HINs) proteins was identified between the six‐rowed and two‐rowed barley cultivars that might contribute to the differences in seed hardness. This suggests that differences in protein profiles can provide a useful tool for examining more complex traits and identify novel protein marker for crop improvement. In recent studies, we have combined shotgun proteomics and cell biological studies of barley seeds to unravel the spatio‐temporal expression and subcellular localization of hordoindoline across development in barley endosperm (Ibl *et al.*, 2018; Shabrangy *et al.*, 2018).

Stress is a key limiting factor that impairs the growth and yield of agricultural crops. A stressful condition (biotic and abiotic) often leads to delayed seed germination, reduced plant growth and decreased crop yield. The role of proteins in plant stress response is crucial, since proteins are directly involved in shaping novel phenotype by adjusting physiological traits to the altered environment (Chaturvedi *et al.*, 2015; Ghatak *et al.*, 2017a; Jegadeesan *et al.*, 2018; Komatsu *et al.*, 2013). Nouri and Komatsu reported that a number of subcellular localized proteins, including ion/water transporters, reactive oxygen species scavengers and proteins related to signalling and transcriptional regulation, are involved in stress tolerance (Nouri and Komatsu, 2013). Recently, Ghatak and co‐workers demonstrated tissue‐specific (root, seed and leaf) protein regulation in pearl millet under drought stress. Protein candidates such as heat shock proteins (HSPs), storage proteins and late embryogenesis abundant (LEA) showed increased levels in seeds. Further, germin‐like protein (GLP5), annexin, showed enhanced abundance under drought stress in root tissue. Moreover, signalling proteins such as GTP binding protein, leucine‐rich transmembrane protein kinase, calreticulin, calnexin, 14‐3‐3 protein and phosphoinositide‐specific phospholipase C (PI‐PLC) also showed increased levels under stress conditions. Upon validation, these tissue‐specific protein candidates can be an aid to design genetically engineered stress‐tolerant crop plants and they can also be employed for marker‐assisted breeding (MAB) (Ghatak *et al.*, 2016). Similarly, two wheat varieties adapted to different environmental conditions (drought tolerant and sensitive) were quantitatively evaluated to identify inherent differences in protein expression patterns and variety‐specific effect of abscisic acid (ABA) on the root proteome (Alvarez *et al.*, 2014). The tolerant wheat variety in this study had a significantly higher number of ABA‐responsive and ABA‐induced proteins that can play an important role in drought adaptation (Alvarez *et al.*, 2014).

## Metabolomics: rapid readout for gene activity and functional gene discovery

Metabolites can be viewed as an end product of gene activity that defines the biochemical phenotype of an organism (Ghatak *et al.*, 2018; Weckwerth, 2003). High‐throughput metabolomics is an important area in the field of ‘omics’ technologies because it helps to unravel the complexities of the genotype–environment–phenotype relationship and provides information about phenotypic plasticity, which is not predictable from genome sequence information (Weckwerth, 2011). Especially, the application of metabolomics in the field allows the rapid analysis of intraspecific metabolic variation depending on the environmental conditions (Baker *et al.*, 2006; Hoehenwarter *et al.*, 2008; Scherling *et al.*, 2010; Steinfath *et al.*, 2010). Metabolomics is also sensitive enough to unravel silent plant phenotypes (Weckwerth *et al.*, 2004b; Weckwerth and Morgenthal, 2005). Thus, metabolite biomarkers provide a unique chemical fingerprint for trait phenotypes that can be useful for precision breeding. For these reasons, metabolites are increasingly used for predicting phenotypic properties. Recently, metabolic quantitative trait loci (mQTLs) identified varying regulation of metabolism across tissues. Tissue‐specific accumulation of metabolites (primary and secondary metabolites) is of importance for the survival and adaptation of the plant species in changing climatic conditions (Gong *et al.*, 2013). mQTL mapping in rice flag leafs and germinating seeds led to the identification of 44 and 16 potential mQTL ‘hotspots’, respectively (Gong *et al.*, 2013).

Application of metabolomics in plant breeding and identifying stress marker for crop improvement has been previously reviewed (Fernie and Schauer, 2009; Ghatak *et al.*, 2018; Weckwerth, 2011). Over the century, the phenomenon of heterosis is a major focus in plant breeding in order to have improved yield from hybrid progeny compared to either homozygous parent (Schnable and Springer, 2013). The study undertaken by Goff derived a metabolic model for multigenic heterosis; here, it was hypothesized that hybrid progeny exhibit modified protein synthesis and metabolism that increases energy efficiency within the seedling for superior phenotypic performance (Goff, 2011). Furthermore, Lisec and co‐workers determined the association between heterosis and metabolism in maize, to predict hybrid performance using metabolite profiles of both hybrid and their parental lines. In this study, it was revealed that the roots of hybrid maize seedlings exhibit differential metabolomes compared to the parents. Moreover, a negative correlation was established between the plant biomass and selected metabolites from hybrids when compared to inbred parent lines (Lisec *et al.*, 2011).

## Metabolic GWAS: understanding the genetic basis of metabolome dynamics for germplasm improvement

A combination of genome‐wide association studies (GWAS) with metabolomics has emerged as a powerful forward genetics strategy to dissect the genetic and biochemical bases of crop plants (Luo, 2015). Utilization of these intermediate traits (metabolites), which are related to biochemical and physiological status of the plant, provides an extra benefit for the global identification of genetic determinants across the huge diversity of intraspecific variation. This concept is accelerated by the development of high‐throughput mass spectrometry‐based (GC‐MS/LC‐MS) analytical platforms and genome sequencing technologies (Fang *et al.*, 2019).

mGWAS was initially applied in the model species *Arabidopsis thaliana* to identify the effects of widespread genetic variants on metabolic diversity across natural populations (Chan *et al.*, 2010; Fu *et al.*, 2009; Keurentjes, 2009). This was then extended to a number of crop species (Luo, 2015). A common feature in the genetic make‐up of metabolism is the presence of ‘hotspots’ or ‘hotspot region’ of major genes/genome regions determining the natural variation in large sets of primary and secondary metabolites (Knoch *et al.*, 2017). Recently, Li and co‐workers identified 65 primary metabolites that were quantified in four different tissues that showed clear tissue‐specific patterns. Three hundred and fifty quantitative trait loci (QTLs) for these metabolites were examined, which were distributed unevenly across the genome and included two QTL hotspots (Li *et al.*, 2019).

Precisely designed mGWAS based on individual metabolite content and ratios under different conditions will not only contribute to the understanding of metabolic diversity under constantly changing environment, but also lead to the identification of key regulators for stress responses (biotic and abiotic). However, two main questions still remain open in the mGWAS analysis (i) How to dissect metabolite pathways and characterize gene function in post‐transcription, post‐translation and epigenetic condition? (ii) mGWAS is based on the ratio of each individual metabolite under different conditions, is this approach dynamic enough to dissect mechanisms underlying metabolic responses to environmental stimuli?

## Phenomics: the virtue of plant phenotyping

The phenotypic outcome depends upon single or multiple genes and their interaction with the environment. Phenomics is the systematic study that involves the gathering of phenotypic data from multiple levels of the organization and progress towards the more complete characterization of the holistic phenotype (Dhondt *et al.*, 2013). The main aim of phenomic platforms is to speed up the process of phenotyping using highly automated robotic transportation system, sensors, imaging systems and computational power.

Rapid and precise phenotypic assessment is of utmost importance to link genotype, agronomic trait and plant function. This assessment can analyse complex traits, which is relevant for plant selection and also provides an explanation for the response of a given genotype in a specific environment (control and stress) (Furbank and Tester, 2011). It can operate at different levels of resolution and dimension (i.e. from molecular cell to the whole plant). Furthermore, it can also evaluate the outcome obtained from genome‐edited crop plants (i.e. mutagenesis, genetically modified organism or CRISPR/Cas9; Blum, 2014; Stutzel *et al.*, 2016). These assessments provide knowledge in the context of a research setting, and for the breeding community, this can be misleading information because the controlled nature of many phenotyping platforms cannot fully replicate the ‘real’ environmental factors (i.e. field condition) that influence complex traits. Moreover, the conventional procedure of phenotyping is not suitable for large‐scale application, and its precision is also not high enough. Furthermore, manual phenotyping of approximately 200 plants is technically impractical. Therefore, in this light of direction, it is important to incorporate extensive high‐throughput field phenotyping platforms to support the breeding programme in order to enhance genetic gains with justifiable cost (Araus and Cairns, 2014). The choice of phenotyping under controlled versus field environment largely depends on the purpose of phenotyping and trait of interest along with the consideration of the logistic tools to collect the information, e.g. for the measurement of high atmospheric CO_2_ levels in the field (Cobb *et al.*, 2013; Gleadow *et al.*, 2013).

High‐throughput phenotyping (HTPPs) is majorly based on remote sensing; it is a nondestructive and noninvasive approach. It is based on the principle of gathering information provided by visible/near‐infrared and far‐infrared radiation emitted by the crops. It is the most commonly used approaches in crop phenotyping. The devices used for phenotyping include multispectral, hyperspectral, fluorescence, thermal sensors and imagers (RGB colour cameras). Extensive literature is available with detailed information regarding the use of these devices and advancements in field phenotyping (Araus and Cairns, 2014; Araus *et al.*, 2018; Deery *et al.*, 2016; Fahlgren *et al.*, 2015; Pauli *et al.*, 2016; Rebetzke *et al.*, 2016). Recently, Walter and co‐workers divided phenotyping into four categories: (i) Imaging (RGB: red‐green‐blue): for measuring size, morphology, growth or architecture of the plant or their canopies; (ii) Thermal imaging: based on the indicators such as stomatal transpiration or water status; (iii) Spectral reflectant/fluorescence: for investigating leaves pigments, biochemical and biophysical processes; (iv) Root phenotyping: architecture and physiology of the root system. The amount of data one gathers using such devices can be staggering (Walter *et al.*, 2015). Furthermore, bioinformatics tools, multivariate statistical methods and pattern analysis are required to extract information from these complex phenotyping experiments properly and concisely (Furbank and Tester, 2011). Several publicly accessible bioinformatics resources are available that can allow plant breeders to explore a multitude of experiments and identify traits that are of importance for future agricultural developments, such as yield stability. This information is also available for a variety of crops from different environments and climates. These public repositories are interactive domains where all deposited data can be viewed graphically and downloaded for independent analysis, e.g. DRYAD (http://datadryad.org/); Phenome Networks database (http://phnserver.phenome‐networks.com/); PHOTOSYNQ (https://photosynq.org/); International plant phenotyping network (IPPN) (https://www.plant‐phenotyping.org/IPPN_home); and European Infrastructure for Multi‐scale Plant Phenomics and Simulation (EMPHASIS) (https://emphasis.plant‐phenotyping.eu/). However, in the current scenario, phenotypic data should be cross‐referenced to the other plant resources such as gene banks, specific genomic information, syntenic, genetic and physical maps, SNP marker, metabolomics and proteomics databases. Eventually, such cross‐reference between phenotype and genomic information can be exploited for crop improvement. Moreover, integrating speed breeding platforms (Watson *et al.*, 2018) with HTPPs can further accelerate gene discovery and characterize the effect of specific genes for plant growth and yield.

Early stress detection is always challenging for phenotyping, and several techniques such as chlorophyll fluorescence, visible and infrared spectroscopy and hyperspectral imaging are being used in the research sector (i.e. controlled environment; Fang and Ramasamy, 2015; Mutka and Bart, 2015). However, to use or establish such techniques under field conditions at canopy level is a technological challenge. Recently, solar‐induced chlorophyll fluorescence (SIF) technique has gained much attention due to its improved sensors (hyperspectral imaging at minimum 1nm wavelength range with incident light sensor) and algorithm. This technique allows the global assessment of vegetation physiology, particularly monitoring crop photosynthesis on global scales (Guanter *et al.*, 2012; Pinto *et al.*, 2016). Recently, this technique was used in the large canopy of commercially grown oranges (*Citrus sinensis* L. cv. Powell) in Spain to track photosynthesis at different phenological and stress stages throughout the season and to support its application in the context of precision breeding (Zarco‐Tejada *et al.*, 2016). Similarly, remote sensing techniques can further be used to assess the stress parameter with the help of distributing phenotypic sites in the fields.

## PANOMICS platform and systems modelling for germplasm improvement

The aim of integrating all these different technology platforms, the PANOMICS approach, is to combine different data types (genome, RNA, proteins, metabolites, phenome) and generate models that can be used to predict complex traits (Weckwerth, 2011; Weckwerth, 2019). Thorough integration of phenomics and environmental information with genomics, transcriptomics, proteomics and metabolomics will also provide a better understanding of the terroir‐phenotype dependency at a molecular level. Integration of multi‐omics data can also reduce false positives generated from single data sources for the genotype–phenotype prediction (Ritchie *et al.*, 2015). Here, data integration is the challenging task, because the diversity and different scales of the data generated from these various high‐throughput technologies (machine sensitivity, error rate, data structure) make their combination difficult. Data integration is mainly and widely done with three approaches: (i) different ‘omics’ data set are analysed in isolation in order to identify the key ‘features’ of each analysis. Upon identification of the significant features, the information is networked together to obtain overall model pathways of the system. More convenient is the sequential extraction and analysis of metabolites, proteins, transcripts and other molecular components from the same sample (Weckwerth *et al.*, 2004a; Valledor *et al.*, 2014). With respect to data integration this is the most straightforward and precise strategy for the application of statistical tools to reveal multivariate pattern and correlations in the data. This approach is widely used for the assessment of a biological system under stress condition. (ii) For integrated data analysis approach, specialized tools are available to merge multi‐omics data set prior to any analysis and interpretation (Kuo *et al.*, 2013; e.g. tools such as COBRA, Mapman, MetaboAnalyst, mixOmics, COVAIN, SIMCA). One such example is orthogonal two‐way projection to latent structures (O2PLS) and its variant OnPLS, these tools were developed to identify systematic variation that is common between two omics data set, and the obtained output is much easier to interpret and the outliers are quickly detected (Trygg, 2002; Trygg and Wold, 2002). It is highly used in studies where more than one environmental perturbation is involved. In addition, there are several ‘one‐click’ platforms available that can assist in statistical analysis (uni‐ and multivariate analyses) of the integrated omics data set along with pathway and gene ontology analysis (e.g. COVAIN packages; Sun and Weckwerth, 2012). (iii) Systems modelling and simulation techniques are based on mathematical equations; these are valuable tools for understanding and even predicting the causal–functional relationship of the complex biological systems in relation to its environment (Weckwerth, 2019). Such integration methods highly rely on a well‐defined qualitative or even quantitative structure of the system being investigated in order to compare new experimental findings. Such an understanding of the system is often based on having comprehensive, genomics, transcriptomics and/or metabolomics data (Weckwerth, 2019). These modelling systems may incorporate dynamic/kinetic models that solve systems with differential, or partial differential or inverse stochastic Lyapunov matrix equations (Weckwerth, 2019), or flux‐balance models (Orth *et al.*, 2010). Interestingly, almost all of these systems modelling approaches are attached to metabolic reactions from genome‐scale metabolic reconstruction and high‐throughput metabolomics data. This emphasizes that in systems modelling, metabolomics plays a key role in multi‐omics data integration because it provides tools for a rapid and holistic quantitation of important parameters of the system. System modelling cannot be performed without quantitative inputs, and likewise, system models cannot be verified without quantitative output. Hence, metabolomics serves both, a quantitative input and output. Quantitative proteomics can also be used, but it is not as close to the observed phenotype as the metabolome. Therefore, one of the central challenges for the systems modelling approach is the collection of quantitative reference data from genome, transcriptome, proteome and metabolome (Pinu *et al.*, 2019). One such example for the integrated pipeline is the COVRECON strategy that shows the systematic linkage of genome‐scale metabolic reconstruction, multi‐omics measurement of the system and the inverse Lyapunov matrix equation for functional prediction. This pipeline can be widely used for a variety of complex systems (Weckwerth, 2019).

Besides the aforementioned techniques, recently deep learning (DL) is gaining momentum in multi‐omics data integration (Lecun *et al.*, 2015). Deep learning (DL) is a subdomain of machine learning (ML), which has emerged as a powerful approach, which can encode and model many forms of complex data (e.g. numeric, text, audio, and image) in both supervised (e.g. biomarker identification) and unsupervised (e.g. anomaly detection) manner (LeCun, Bengio, and Hinton, 2015). Here, ‘deeper’ neural networks provide a hierarchical representation of the data by utilizing various convolutions. This allows larger training and learning process, which provides higher performance with precision. A prominent difference between deep learning (DL) and traditional machine learning (ML) such as artificial neural networks (ANNs) includes DL models’ capacity to learn and fit raw data through representation at multiple levels of abstraction or hidden layers (Lecun *et al.*, 2015). This essentially produces more refinement of the representation of observed patterns in upper layers, in contrast to the ANNs, which only contain three layers: input, hidden and output (Ching *et al.*, 2018). Novel DL architectures are continuously developed (Angermueller *et al.*, 2016; Ching *et al.*, 2018; Lecun *et al.*, 2015; Min et al., 2017; Tran *et al.*, 2017), which includes deep neural networks (DNN), convolutional neural networks (CNNs), recurrent neural networks (RNNs) and auto‐encoders (Pérez‐Enciso and Zingaretti, 2019). There are multiple examples for applications of these newly developed architectures in plant biology (Gao *et al.*, 2018; Ghosal *et al.*, 2018; Wang *et al.*, 2009; Washburn *et al.*, 2019). Deep learning (DL) has met popularity in numerous applications dealing with raster‐based data (e.g. video, images), which has brought paradigm shift in image‐based plant phenotyping as a nondestructive method that can provide major advantages to the plant breeders, pathologists, physiologists with an opportunity to search large data sets to discover patterns and govern discovery by simultaneously looking at a combination of factors instead of analysing each feature (trait) individually (Singh *et al.*, 2018). This was previously a major bottleneck because the high dimensionality of individual images (coupled with the huge number of such images) makes them extremely difficult to analyse through classical techniques (Singh *et al.*, 2015). A wide range of DL architectures has been used in plant phenotyping, including DCNN (Pound *et al.*, 2017), RCNN and ResNet (Fuentes *et al.*, 2017), SegNet (Aich and Stavness, 2017) and AlexNet (Mohanty *et al.*, 2016). DL architectures have performed well on a broad range of plant phenotyping tasks, such as plant identification based on leaf vein patterns (Grinblat *et al.*, 2016), leaf counting (Ubbens *et al.*, 2018), panicle segmentation (Xiong *et al.*, 2017), and plant recognition (Šulc and Matas, 2017) and others. Deep learning models have also been used for crop yield prediction, considering a case study from Syngenta Crop Challenge 2018, which has released several large data sets that recorded the genotype and yield performances of 2267 maize hybrids planted in 2247 locations between 2008 and 2016 to predict the yield performance in 2017. Deep neural network (DNN) approach was used for modelling and solution techniques. Khaki and Wang developed a model that has a superior prediction accuracy, with a root‐mean‐square error (RMSE) being 12% of the average yield and 50% of the standard deviation for the validation data set using predicted weather data. They also performed feature selection that successfully decreased the dimension of the input space without a significant drop in the prediction accuracy. Hence, this model significantly surpassed other popular methods such as Lasso, shallow neural networks (SNN) and regression tree (RT) (Khaki and Wang, 2019). However, the major limitation of the proposed model was its black box property, which is shared by many machine learning methods. Although the model captures G × E interactions, its complex model structure makes it hard to produce testable hypotheses that could potentially provide biological insights. Hence, environmental factor can be employed for feature selection approach to make the model less complicated (Khaki and Wang, 2019).

Visual exploration of the network models is also an integral part of data integration. Nowadays, it is easily done through network manipulation software like Cytoscape (Shannon *et al.*, 2003). Its basic visualization feature allows the first level of network understanding. For example, grouping nodes by interaction level provides information about the hierarchy of regulatory events. This visualization approach revealed two mutually inhibiting groups of genes during lateral root development in *Arabidopsis thaliana* (Lavenus *et al.*, 2015).

## Integrating GWAS with the PANOMICS platform explains more phenotypic variance

In order to predict genetic risk factor for agronomically important traits (i.e. yield and growth rate) in plants, it is of utmost importance to understand and gather information about both the specific loci that underlie a phenotype, and the genetic architecture of a trait (Korte and Farlow, 2013). Moreover, Mendel already postulated the existence of ‘internal factors’ that are passed onto the next generation (Lander and Botstein, 1989). Therefore, understanding of genotype and phenotype relationships is of major interest and importance. Advances in high‐throughput and high‐dimensional genotyping and phenotyping technologies enabled the discovery of potential links between genotypes and phenotypes using the principles of genome‐wide association studies (GWAS). Here, allelic polymorphisms in the genome and its corresponding phenotypes are screened systematically to reveal their correlation with phenotypic traits. In most cases, linkage disequilibrium and accumulation of single nucleotide polymorphisms (SNPs) in a specific genomic region are harnessed to find a potential relationship between genome and phenome (Nordborg *et al.*, 2002). However, this approach does not go beyond an associative or correlative relationship with no proof of causality. For the identification of causal chains from genes to phenotypes, the PANOMICS platform is inevitable. Beló and co‐authors demonstrated the full workflow in maize starting with GWAS and finally identifying the causal single gene for increased oleic acid in maize seeds (Belo *et al.*, 2008). QTL mapping has also proven to be a powerful method to identify regions of the genomes that co‐segregate with the given trait either in the biparental population such as double haploids, F_2_ generation or recombinant inbred lines (RILs). However, these mapping populations are products from few cycles of recombination events, limiting the resolution of genetic maps that often do not represent germplasm that is actively used in breeding programmes. By contrast, GWAS overcome these limitations and provide greater resolution for identifying genes potentially responsible for variation in a quantitative trait (Doerge, 2002).

GWAS have been successfully carried out in many crop species such as maize, rice, wheat, sorghum and foxtail millet (see Table 1). These successful examples are also extensively reviewed by Huang and Han (2014); Ogura and Busch (2015). Huang and co‐workers genotyped 517 rice landraces (*Oryza sativa* indica subspecies) with the identification of ~3.6 million SNPs and phenotyped 14 agronomic traits. Interestingly, the obtained result only explained ~36% of phenotypic variances and the complex genetic architecture of these traits (Huang *et al.*, 2010b). Overall, the results presented in Table 1 show that current GWAS published data can actually explain only ~40% of phenotypic variance; that is, each SNP identified is only explaining a small percentage of variance. Therefore, we anticipate that in order to explain and understand the remaining ~60% of phenotypic variance, it is extremely important to integrate GWAS with the PANOMICS platform leading to complementary genome‐based high‐throughput data sets such as transcriptomics (eQTLS), proteomics (pQTLS) and metabolomics (mQTLS). This integration can lead to the identification of not only novel genes but also functional pathways underlying complex traits. Moreover, these integrative studies will also have several advantages which can be complementary to SNP–trait association studies: (i) they can reflect variation in both genetic and epigenetic regulatory component and (ii) they can provide additional evidence to fine map QTL. In this line of light, a combination of mGWAS with eQTL led to the identification of novel biochemical insights of maize kernels and also identified two metabolite features associated with kernel weight that can be used as biomarkers for genetic improvement in maize (Wen *et al.*, 2014). Recently, in situ eco‐metabolomics in combination with SNP enrichment and metabolic modelling revealed potential biochemical adaptation processes of *Arabidopsis thaliana* to the natural habitat and micro‐environment (Nagler *et al.*, 2018). A conclusion from this study is that every location and its microhabitat creates a unique phenotype. This suggests that locally selected and developed cultivars may be superior to seed stocks produced and distributed globally.

**Table 1:** GWAS studies and phenotypic variance

Crops	Species	Cultivars studied	GWAS studies (No. of Traits)	SNP’s identified	Phenotypic variance	References
Arabidopsis	*Arabidopsis thaliana*	192	107	~216 130	~20%	Atwell *et al. *(2010)
Rice	*Oryza sativa* L.	517	14	~3.6 million	~36%	Huang *et al. *(2010b)
Rice	*Oryza sativa* L.	20	NA	~160 000	NA	McNally *et al. *(2009)
Rice	*Oryza sativa ssp. japonica*	176	4	~426 337	~30%–35%	Yano *et al. *(2016)
Rice	*Oryza sativa ssp. Japonica*	193	5	~1713	~20%–40%	Reig‐Valiente *et al. *(2018)
Rice	*Oryza sativa* L.	369	19	~71 710	~30%–40%	Begum *et al. *(2015)
Wheat	*Triticum aestivum* L.	723	23	52 303 DArT‐seq marker	~30.20%	Liu *et al. *(2017)
Wheat	*Triticum aestivum* L.	105	9	~15 430	~10.86%–20.27%	Wang *et al. *(2017)
Bread wheat	*Triticum aestivum* L.	163	13	~20 689	~20%	Sun *et al. *(2017)
Bread wheat	*Triticum aestivum* L.	93	9	~16 383 silico DArTs marker	~20%	Mwadzingeni *et al. *(2017)
Spring wheat	*Triticum aestivum* L.	194	12	~3254	NA	Turuspekov *et al. *(2017)
*Aegilops tauschii*	*Triticum aestivum* L.	322	29	~7185	~8%–23%	Liu *et al. *(2015)
Barley	*Hordeum vulgare* L.	122	14	~9680	~30%–40%	Hu *et al. *(2018)
Barley	*Hordeum vulgare* L.	1420	9	~5398	~35%–40%	Sharma *et al. *(2018)
Barley	*Hordeum vulgare* L.	224	5	~1536	~20%–30%	Pasam *et al. *(2012)
Barley	*Hordeum vulgare* L.	223	17	~816 DArT, SNP and SSR	~0.6%–3.8%	Varshney *et al. *(2012)
Soybean	*Glycine max*	169	3	~3780	~9%–15%	Contreras‐Soto *et al. *(2017)
Sorghum	*Sorghum bicolor *(L.) *Moench.*	971	2	∼265 000	~40%	Morris *et al. *(2013)
Sorghum	*Sorghum bicolor* L*.*	245	5	~85 585	~15%–20%	Li *et al. *(2018)
Maize	*Zea mays* L.	368	1	~559 285	~4%–7%	Li *et al. *(2016)
Maize	*Zea mays* L.	508	4	~543 641	~10%–15%	Cui *et al. *(2016)
Maize	*Zea mays* L.	346	10	~60 000	~3%–7%	Farfan *et al. *(2015)
Maize	*Zea mays* L.	289	3	~56 110	~32%	Riedelsheimer *et al. *(2012)
Maize	*Zea mays* L.	350	9	~56 110	~15%–20%	Xue *et al. *(2013)
Maize	*Zea mays* L.	513	17	~0.5	~40%	Yang *et al. *(2014)
Foxtail millet	*Setaria italica*	916	47	~845 787	NA	Jia *et al. *(2013)
Tomato	*Solanum lycopersicum*	163	19	~5995	~30%–40%	Sauvage *et al. *(2014)
Cassava	*Manihot esculenta* Crantz	158	11	~349 827	~30%–40%	Zhang *et al. *(2018)
Peanut	*Arachis hypogaea* L.	300	50	~154 SSR, 4597 DArTs marker	~30%–40%	Pandey *et al. *(2014)

## PANOMICS‐guided genome editing for precision breeding to enhance climate resilience and nutritional value of germplasm

Genome editing technologies enable precise manipulation of specific genomic sequences. With the help of these approaches, a point mutation (deletion or insertion), gene knockouts, activation or repression of genes and epigenetic changes are possible (Kamburova *et al.*, 2017). Such technologies rely on sequence‐specific nucleases (SSNs), and with the help of molecular tools, DNA double‐strand breaks (DSBs) are created at the desired location within the genome. In contrast to the transgenic approach, which leads to random insertions generating random phenotypes, genome editing methods generate defined mutants, thus becoming a potent tool for functional genomics and crop breeding (Kamburova *et al.*, 2017; Malzahn *et al.*, 2017). First‐generation genome editing technologies include several sequence‐specific nucleases such as meganucleases, zinc finger nucleases (ZFNs) and TAL effector nucleases (TALENs), which rely on just one or two non‐elite genotypes that are susceptible to regeneration from plant tissue culture and transformation. However, these techniques involve tedious procedures to achieve target specificity; they are also labour‐intensive and time‐consuming. In contrast, the second‐generation genome editing techniques include pol/Cas9; it has a simple design and straight forward execution methodologies that involve guide RNA (gRNA) of about 20 nucleotides complementary to the DNA stretch within the target site of the gene, and hence, it is more time and cost‐effective (Voytas, 2013). Most recently, the CRISPR/Cas‐mediated genome editing (CMGE) approach has become a method of choice and it is being extensively used to edit plant genomes compared to ZFNs/TALENs (Jaganathan *et al.*, 2018). This method has been adopted in nearly 20 crop species for various traits such as yield improvement, biotic and abiotic stress. For more details, please refer to many specialized review articles that provide insight of the methodology as well as proof of concept studies that determine the successful application of CRISPR/Cas (Belhaj *et al.*, 2015; Jaganathan *et al.*, 2018).

**Figure 3:**
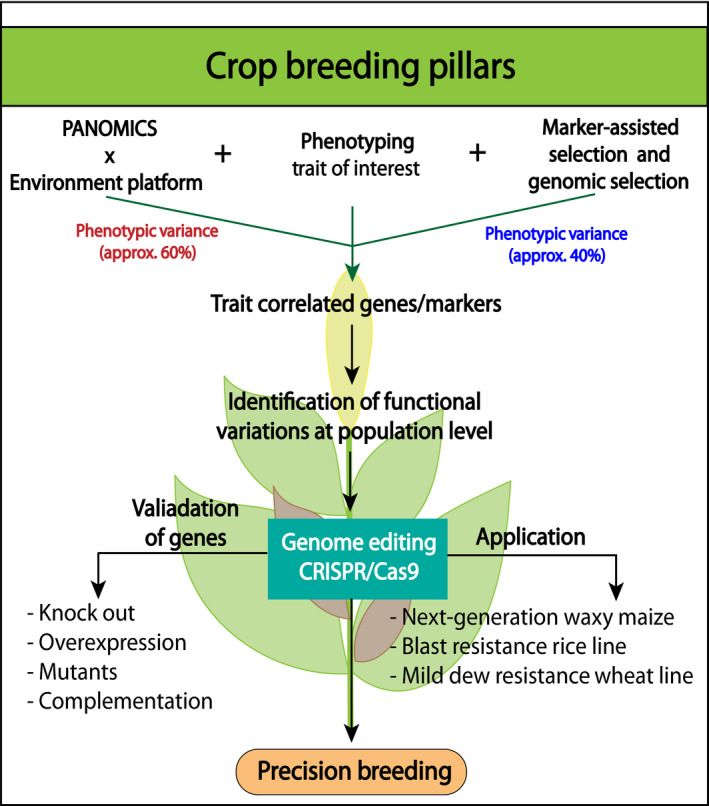
Crop breeding pillars for precision breeding strategy. Currently, agronomically important genes are identified using marker‐assisted selection breeding (MAS) and genomic selection, which only provides ~40% of the phenotypic variance. In future, integration of PANOMICS platform will not only enhance the identification of remaining ~60% of phenotypic variance but also support the identification of agronomical trait correlated to the genes in the most rapid and effective manner to support precision breeding.

The development of precision breeding will require closer integration of the PANOMICS platform and genome editing tools. Here, we propose the enhancement of two‐way communication between multi‐omics and genome editing tools (Figure 3). As discussed, advances in phenotyping and multi‐omics technologies generated large‐scale data known as ‘Big Data’ which have provided sufficient power to elucidate a large number of trait‐specific genes. Hence, it is of utmost importance to functionally validate the candidate of interest. In this light, technological and biological limitations are now at the forefront of research interest because functional genetics is laborious and several scientific techniques (robust tissue culture methods) do not have an impact. Due to these shortcomings, many candidate genes are not followed or functionally validated. Therefore, harnessing the power of genome editing provides a unique opportunity to understand a genetic basis at the population level for different phenotypic groups by parallel analysis of multiple target genes for loss of function and associated trait alteration (Figure 3). Applying this methodology for crop improvement would be advantageous considering the relative resources (time and money) as well as for obtaining precision in trait optimization for the desired phenotype (such as yield, nutritional value and plant fitness). Integrating genome editing technique with speed breeding approach can further facilitate validation of incorporated gene without in vitro manipulations. Moreover, phenotyping can also be performed in subsequent generations, allowing identification of the trait that can further be exploited (Hickey *et al.*, 2019). These outcomes may enhance the development of new markers that can be employed routinely in the breeding process, thereby securing food productivity. However, public consent for genome modification in agriculture is important for proper exploitation of this methodology in order to support developing regions across the globe (http://www.fao.org). Several public institutes such as ICRISAT (International Crop Research Institute for Semi‐Arid Tropics), which belongs to the domain of CGIAR (Consultative Group on International Agriculture Research), have been accelerating use of genome editing tools (specifically CRISPR/Cas) for enhancing crop production and support smallholder farmers (https://www.icrisat.org/icrisat‐and‐corteva‐agriscience‐agriculture‐division‐of‐dowdupont‐collaborate‐for‐sharing‐advanced‐breeding‐technologies‐to‐improve‐crops‐that‐feed‐millions/). This kind of initiative will accelerate the process of bringing healthier legumes (such as chickpea and pigeon pea) to the consumers (Khoury *et al.*, 2014). In Table 2, we have assembled the nutritional value of major staple food crops and their available germplasm collections. Significant intra‐ as well as interspecific differences can be found in the overall protein, carbohydrate, fat and fiber contents as well as in the mineral and vitamin compositions. These important values can be determined over large germplasm collections with sequenced genomes, diversified and optimized by the proposed precision breeding strategies. Accordingly, a major approach of PANOMICS that meets germplasm collections will be the improvement of the nutritional value of these crops in the balance of stress resistance and productivity. These common goals can only be addressed by combining multi‐omics characterization, for example the production of proteins, carbohydrates, fats, vitamins and minerals, and genomic selection. Furthermore, it can also enhance the production of other cereal crops such as wheat, sorghum and pearl millet for more elite lines and also improve the existing germplasm for higher stress tolerance and nutritional value. Most importantly, the implementation of precision breeding evidently depends upon the creation of improved infrastructure and ethical norms, as well as the establishment of more powerful computational tools on a routine basis.

**Table 2:** Nutritional value of major staple food crops per 100 g and their available whole‐genome sequence germplasm collections

Staple food crop	Botanical Name	Protein (g)	Fat (g)	CHO (g)	Dietrary fiber (g)	K (mg)	Mg (mg)	P (mg)	Fe (mg)	Zn (mg)	Vit C (mg)	Vit B6 (mg)	Literature	Germplasm collections
Wheat	*Triticum aestivum*	9.03–12.25	1.95–3.48	72.6–76.6	0.79–0.93 (13.1 whole grain)	394–400^1^	117–150^1^	323–480^1^	2.6–3.71	2.0–2.96	0–1.2	0.191–0.5	Anglani (1998), Plaza *et al*. (2003), USDA^2^	Liu *et al.* (2017), Wang *et al.* (2017), Sun *et al.* (2017), Mwadzingeni *et al.* (2017), Turuspekov *et al.*(2017)
Rice	*Oryza sativa*	5.95–7.23	1.42–2.78	76.48–80.13	2.4–4.6	76–289	35–112	98–337	0.35–1.98	0.8–2.45	0	0.436–0.736	USDA^3^, ^4^	Huang *et al.* (2010a), McNally *et al.* (2009), Yano *et al.*(2016), Reig‐Valiente *et al.* (2018), Begum *et al.* (2015)
Maize	*Zea mays*	6.93	3.86	76.85	7.3	315	93	272	2.38	1.73	0	0.37	USDA^5^	Yang *et al.*(2014), Cui *et al.* (2016), Xue *et al.* (2013), Riedelsheimer *et al.*(2012), Farfan *et al.*(2015), Li *et al.* (2016)
Oat	*Avena sativa*	13.15	6.52	67.7	10.1	362	138	410	4.25	3.64	0	0.1	USDA^6^	Bekele *et al.* (2018)
Pearl Millet	*Pennisetum glaucum*	11	4.2	73	1.2 to 6.5	195	114	285	3–8	1.7–3.1	0	0.384	Malik (2015), USDA^7^	Varshney *et al.*( 2017b)
Sorghum	*Sorghum bicolor*	10.4–10.62	1.9–3.46	72.6–72.09	1.6–6.7	363	165–171	222–289	3.36–4.1	1.6	0	0.443	Nambiar *et al.* (2011), USDA^8^	Morris *et al.* (2013), Li *et al.* (2018)
Barley	*Hordeum vulgare*	10.42–12.48	1.04–2.3	73.48–77.08	14.6–17.3	281–452	133	264	2.25–3.6	2.77	0	0.318	USDA^9^, ^10^	Hu *et al.* (2018), Sharma *et al.*(2018), Pasam *et al.*( 2012), Varshney *et al.* (2012)
Soybean	*Glycine max*	36.49^11^–46.8	16.6–19.8	8.2–30.16^11^	9.3^11^	207–1797^11^	133–280^11^	704^11^	4.9^12^–15.7^11^	2.4^12^–4.89^11^	6^11^–10.0^12^	0.377^11^–0.8^12^	Plaza *et al.* (2003), Wilcox and Shibles (2001),^11^ USDA	Contreras‐Soto *et al.* (2017)
Chickpea	*Cicer arietinum*	23.7^13^	4.8^13^–6.0	60.7–61.1^13^	14.8^13^–17.4	870–1215.7	176	226–505.1	4.4–7.72	3.43–4.32	1.34–6.0	0.3–0.55	el‐Adawy (2002), Jukanti *et al.* (2012), Sreerama *et al.* (2012)	Varshney *et al.* (2019)
Pigeonpea	*Cajanus cajan*	7.2^15^–22.6	1.49^14^–3.15	23.88^15^–62.78	1.12–15^14^	552^15^–1392^14^	68^15^–183^14^	127^15^–367^14^	1.6^15^–5.23^14^	1.04^15^–2.76^14^	0^14^–39^15^	0.068^15^–0.283^14^	Solomon *et al.* (2017),^14^, ^15^ USDA	Varshney *et al.* (2017a)
Lentils	*Lens culinaris*	24.63	1.06	63.35	10.7	677	47	281	6.51	3.27	4.5	0.54	USDA^16^	Pavan *et al*. (2019)
Potato	*Solanum tuberosum*	6.9	0.34	83.1	5.9	1001	65	168	1.38	0.54	3.8	0.769	USDA^17^	Uitdewilligen *et al.* (2013)
Yam	*Dioscorea*	1.53	0.17	27.88	4.1	816	21	55	0.54	0.24	17.1	0.293	USDA^18^	Saski *et al.* (2015), Girma *et al.* (2014)

CHO, Carbohydrates; K, Potassium; Mg, Magnesium; P, Phosphorus; Fe, Iron; Zn, Zinc; Vit C, Vitamin C; Vit B6, Vitamin B6; USDA, United States Department of Agriculture.

^1^ Dry matter of grain, grown under field conditions (Anglani, 1998).

^2^ Whole Grain Flower, https://fdc.nal.usda.gov/fdc-app.html#/food-details/168944/nutrients

^3^ Rice, unenriched white flour,https://fdc.nal.usda.gov/fdc-app.html#/food-details/169714/nutrients

^4^ Rice flour, brown, https://fdc.nal.usda.gov/fdc-app.html#/food-details/168898/nutrients

^5^ Corn flour, whole‐grain, yellow, https://fdc.nal.usda.gov/fdc-app.html#/food-details/170290/nutrients

^6^ Oats, raw, https://fdc.nal.usda.gov/fdc-app.html#/food-details/340734/nutrients

^7^ Millet, raw, https://fdc.nal.usda.gov/fdc-app.html#/food-details/169702/nutrients

^8^ Sorghum grain, https://fdc.nal.usda.gov/fdc-app.html#/food-details/169716/nutrients

^9^ https://fdc.nal.usda.gov/fdc-app.html#/food-details/489276/nutrients

^10^ Barley, hulled, https://fdc.nal.usda.gov/fdc-app.html#/food-details/170283/nutrients

^11^ Soybeans, mature seeds, raw, https://fdc.nal.usda.gov/fdc-app.html#/food-details/174270/nutrients

^12^ mg/100 g dry weight (Plaza et al., 2003)

^13^ Legume flour, calculated as % dry matter

^14^ Pigeon pea (red gram), mature seeds, raw, https://fdc.nal.usda.gov/fdc-app.html#/food-details/172436/nutrients

^15^ Pigeon pea, immature seeds, raw, https://fdc.nal.usda.gov/fdc-app.html#/food-details/170025/nutrients

^16^ Lentils, raw, https://fdc.nal.usda.gov/fdc-app.html#/food-details/172420/nutrients

^17^ Potato flour, https://fdc.nal.usda.gov/fdc-app.html#/food-details/168446/nutrients

^18^ Yam, raw, https://fdc.nal.usda.gov/fdc-app.html#/food-details/170071/nutrients

## Concluding remarks and perspectives

The rapid advancement of NGS and high‐throughput phenotyping technology opened the era of ‘Big Data’. The reference genome sequences of various crops, model plants and minor plants are constructed by the strength of technological and analytical progress. Along with numerous reference genomes, genetic and genomic resources have also been enriched by genome‐wide analyses using types of resequencing and genotyping approaches to reveal hidden bridges between genomic variations and diverse phenotypes in plant species. Furthermore, characterization of the germplasm through non‐DNA markers (such as transcripts, proteins and metabolites) will allow one to perform molecular characterization of genotypes, providing the list of candidate genes/gene products that are highly valuable for breeding and engineering stress‐tolerant crops with novel and valuable traits not reachable by classical genome prediction methods. Still, proteomics and metabolomics studies are often regarded as holistic studies due to the fact that not many putative markers are translated into the productive sector. This is because high‐throughput techniques are still emerging and require steady improvement in instrumentation and algorithms. The cost of generating high‐throughput data needs to decrease substantially because it is important to identify relevant genotype‐phenotype associations that are not predictable from the genome sequence. However, metabolomics platforms are nowadays partially cheaper than NGS platforms and have a higher throughput. Metabolite analysis is very important to understand, for example nutritional value of crops, but also stress resistance. Accordingly, the metabolic readout can be a rapid predictor of important traits of large cohorts of samples. Here, we propose the complementation with PANOMICS because these technologies become more and more cost‐effective and will improve genomic prediction. Eventually, the integration of PANOMICS platforms with systems modelling and genome editing techniques will enhance precision breeding and support quick breeding procedures (such as SPEED breeding) with the ultimate outcome of providing the appropriate cultivars for each agroecological scenario.

## Conflict of interest

The authors declare no competing financial interest.

## Author contributions

WW, PC, AG and RKV conceived the conception of the manuscript; PC, AG and WW drafted the manuscript; RKV critically reviewed the manuscript; AG and AB prepared data tables; PC and AG designed the figures. All authors have revised and approved the final version of the manuscript.
